# Protective Effects of the Soluble Receptor for Advanced Glycation End-Products on Pyroptosis during Myocardial Ischemia-Reperfusion

**DOI:** 10.1155/2021/9570971

**Published:** 2021-12-06

**Authors:** Yingming Liu, Xinying Guo, Jie Zhang, Xuejie Han, Hongxia Wang, Fenghe Du, Xiangjun Zeng, Caixia Guo

**Affiliations:** ^1^Cardiovascular Center, Beijing Tongren Hospital, Capital Medical University, No. 1 Dongjiaomin Lane, Dongcheng District, Beijing 100730, China; ^2^Department of Physiology and Pathophysiology, Capital Medical University, No. 10 You An Men Wai Xi Tou Tiao, Fengtai District, Beijing 100069, China; ^3^Department of Geriatrics, Beijing Tiantan Hospital, Capital Medical University, No. 119 South 4th Ring West Road, Fengtai District, Beijing 100070, China

## Abstract

Ischemia-reperfusion injury (IRI) is an inevitable process when reperfusion therapy undergoes in acute myocardial infarction patients, which will lead to cardiac cell death. Many factors have been found to protect the myocardium, one of which was the soluble receptor for advanced glycation end-products (sRAGE) that protected the myocardium from apoptosis and autophagy. However, pyroptosis is also an important form of cell death that occurs during ischemia-reperfusion (I/R), whose critical molecule, NLR family pyrin domain containing 3 (NLRP3), was ever reported to be inhibited by sRAGE; therefore, it is hypothesized that sRAGE may decrease the cardiac pyroptosis induced by I/R. The results showed that sRAGE protected cardiomyocytes from I/R-induced pyroptosis by decreasing the expression level of NLRP3, gasdermin D (GSDMD), interleukin-1*β* (IL-1*β*), and interleukin-18 (IL-18). Meanwhile, the results from primary cultured cardiomyocytes showed that the NF-*κ*B pathway mediated the effects of sRAGE on pyroptosis. Therefore, it is concluded that sRAGE protects the heart from pyroptosis through inhibiting the NF-*κ*B pathway during myocardial ischemia-reperfusion.

## 1. Introduction

Acute myocardial infarction (AMI) accounts for more than one-third of all deaths in developed countries and affects more than 7 million individuals worldwide each year, which might be followed by ischemia-reperfusion injury due to the myocardial cell death caused by the reperfusion therapy applied in the clinic as early as possible [1–8]. These ischemia-reperfusion injuries decreased the benefits from clinic treatment in AMI patients; therefore, decreasing reperfusion injuries were expected to improve the prognosis of AMI patients [2, 9].

During myocardial ischemia-reperfusion injury (IRI), various programs of cell death account for the prognosis of the treatment, which includes apoptosis-, necrosis-, and autophagy-associated cell death [10, 11]. Recently, pyroptosis has been reported to be an important part of cell death in ischemia-reperfusion injury as well [11].

Pyroptosis is a type of programmed cell death accompanied by an inflammatory response, which is mediated by NLR family pyrin domain containing 3 (NLRP3) inflammasome and characterized by the cleavage of gasdermin D (GSDMD) [12–15]. When IRI occurs, the “priming” step of NLRP3 inflammasome will be promoted through the NF-*κ*B pathway, which is crucial for inflammasome activation in cardiomyocytes [16]. Following trigger signals including mitochondrial dysfunction, oxidative stress, Ca^2+^ overload, and K^+^ efflux [12], the procaspase-1 will be activated by interacting with NLRP3 inflammasome via the apoptosis-associated speck-like protein containing a caspase recruitment domain (ASC) bridge [17]. The activated caspase-1 will hydrolyze GSDMD to expose the NH2-terminal (GSDMD-NT), which binds to the cell membrane to form perforations, generating an inflammatory cascade that leads to a loss of membrane integrity, cell swelling, and release of inflammatory cytokines including interleukin-1*β* (IL-1*β*) and interleukin-18 (IL-18) [12–14, 18, 19]. According to the recent literature, it has been reported that the soluble receptor for advanced glycation end-products (sRAGE) attenuated cardiomyocyte hypertrophy by inhibiting RAGE-NF-*κ*B-NLRP3 activation in H9C2 cells [20]. These findings suggest that sRAGE may be a promising factor to suppress pyroptosis through blocking the formation of NLRP3 inflammasome during myocardial ischemia-reperfusion (I/R).

sRAGE is a soluble form of the receptor for advanced glycation end-products (RAGE) that is widely present in tissues and blood [21]. The classical role of sRAGE is to antagonize RAGE-induced inflammatory responses by binding to ligands for RAGE [22]. In recent years, sRAGE was found to be able to antagonize myocardial ischemia-reperfusion injury through inhibiting apoptosis, autophagy, or promoting angiogenesis in the heart to save cardiomyocytes and heart function [23–26].

Therefore, it was hypothesized that sRAGE attenuates myocardial ischemia-reperfusion injury by decreasing myocardial pyroptosis through the NF-*κ*B pathway.

## 2. Materials and Methods

### 2.1. Animals

Male C57BL/6 mice (6–8 weeks of age) were supplied and cultured by the Laboratory of Animal Experiments at Capital Medical University. All experimental operations were approved by the animal care and use committee of Capital Medical University (Beijing, China) and in accordance with the “Guide for the Care and Use of Laboratory Animals” published by the US National Institutes of Health (Revised 2011). The six-week-old mice (20 ± 2 g) were randomly divided into four groups: sham + GFP group, sham + sRAGE group, I/R + GFP group, and I/R + sRAGE group. The recombinant adeno-associated virus (AAV) vector was used to overexpress sRAGE. Two weeks before the ischemia-reperfusion operation, the sham + sRAGE group and I/R + sRAGE group were injected with AAV9-sRAGE virus (Vigene, 9.72*E*13 v.g./ml) at a concentration of 1*E*9 v.g./g via the tail vein and the sham + GFP group and I/R + GFP group were injected with AAV9-GFP virus (Vigene, 2.35*E*13 v.g./ml) at equal viral titre in the same way.

### 2.2. Mouse I/R Injury Model

After the adeno-associated virus had been expressed for two weeks, the experimental ischemia-reperfusion was performed on eight-week-old mice. The experimental I/R model was induced by transient myocardial ischemia for 30 min followed by reperfusion as described previously [27]. Briefly, the mice were anesthetized by inhalation (2% isoflurane), subjected to left thoracotomy, pericardiectomy, and transient ligation of the left anterior descending (LAD) coronary artery by 6–0 silk for 30 min before closure of the thorax followed by reperfusion for 24 hours. The sham surgery was performed without the final ligation of the coronary artery, and other treatments were the same as the I/R group. The animals were euthanatized using a pentobarbital overdose (100 mg/kg) followed by cervical dislocation.

### 2.3. Echocardiography

Mice were anesthetized by intraperitoneal injection of tribromoethanol (0.01 ml/g of body weight, T48402; Sigma-Aldrich) and fixed in the supine position. The high-resolution ultrasound microscopic Vevo2100 Imaging System (FUJIFILM VisualSonics Inc., USA) for small animals was used to perform the M-type ultrasound detection of the heart as described previously [28]. After the instrument started imaging, the probe was rotated to the parasternal long axis view of the heart to capture the image. The heart rate (HR), ejection fraction (EF), fractional shortening (FS), cardiac output (CO), and stroke volume (SV) were measured using Vevo LAB 3.1.1 software.

### 2.4. Measurement of the Myocardial Infarct Size

2,3,5-Triphenyltetrazolium chloride (TTC) staining was performed on the heart of mice immediately after reperfusion for 24 hours. The heart was stained with 1% TTC (Sigma-Aldrich, St. Louis, Mo, USA), which was injected into the coronary artery through the end of the aortic arch. Then, the treated heart was incubated at 37°C for 5 min and fixed in 4% paraformaldehyde for 4 min. The sample was frozen at −20°C for 10 min, cut along the long axis of the sagittal plane of the left ventricle, and photographed under the microscope (Leica, Germany). The white area represented the infarcted section and the red area represented the noninfarcted section. The myocardial infarct area of each group was quantified by ImageJ software and calculated as the percentage of the myocardial infarct area relative to the sagittal plane area of the left ventricle.

### 2.5. Immunohistochemical Staining of Paraffin Sections

The heart was fixed with 10% neutral formalin, embedded in paraffin, and cut into 4 *μ*m slices. Immunohistochemistry was performed according to the procedure of the DAB detection kit (GK600505; GenenTech, Shanghai, China). In conclusion, sections were incubated with rabbit gasdermin D Antibody (1 : 200, 93709, Cell Signaling Technology, USA), rabbit NLRP3 antibody (1 : 200, ET1610-93, HUABIO, China), rabbit caspase-1 antibody (1 : 200, ET1608-69, HUABIO, China), rabbit anti-IL-1beta antibody (1 : 200, ab9722, Abcam, UK), and rabbit anti-IL-18 antibody (1 : 1000, ab223293, Abcam, UK) and detected by a DAB colorimetric detection kit. Then, the nuclei were stained with hematoxylin. Finally, the images were obtained using a digital slide scanner (Pannoramic SCAN, 3DHISTECH, Budapest, Hungary) and analyzed using ImageJ as described previously [29]. The protein contents were quantified by the average grey value (immunostaining intensity) and the percentage of positive area (staining area) of the positive cells [29].

### 2.6. TUNEL Assay

The heart was fixed with 10% neutral formalin, embedded in paraffin, and cut into 4 *μ*m slices. TUNEL assay was performed according to the procedure of the In Situ Cell Death Detection Kit, TMR red (12156792910; Roche, Mannheim, German). In conclusion, sections were incubated with a 500 *μ*l TUNEL reaction mixture at 37°C for 60 minutes. The cytoskeleton was stained by *α*-actinin antibody (1 : 200, A7811, Sigma-Aldrich, USA) at 4°C overnight. The nuclei were stained with DAPI. Photographs were obtained using an ECLIPSE Ni-U fluorescence microscope (Nikon, Japan) at 40x magnification. The number of TUNEL-positive cells and total cells were counted in each field using ImageJ software (NIH). The percentage of TUNEL-positive cells to total cells was calculated.

### 2.7. Culture and Treatment of Neonatal Rat Ventricular Cardiomyocytes

Sprague-Dawley (SD) rats born within 24 hours of both sexes were supplied by Vital River Laboratory Animal Technology Co. Ltd. (Beijing, China). The primary neonatal cardiomyocytes were isolated as described previously [30]. Transfection with the recombinant adenovirus which expresses GFP or a sequence of the extracellular domain of human RAGE into the primary neonatal cardiomyocytes of different groups at least 24 hours before I/R treatment. When the cardiomyocytes were subjected to ischemic treatment, the complete medium was replaced with “ischemic buffer” [30]; then, the 6-well plates were placed in 37°C, 1%O_2_, 5% CO_2_, and 94% N_2_ tri-gas incubators. After 2 hours of ischemia, the plates were washed twice with phosphate buffer saline (PBS) (SH30256.01, HyClone, USA) and then incubated with Dulbecco's modified Eagle's medium/nutrient mixture F-12 ham (DMEM-F12) (D8437, Sigma-Aldrich, USA) at 37°C, 5% CO_2_, and 21% O_2_ for 24 hours of reperfusion.

### 2.8. RNA Isolation and Quantitative Real-Time RT-PCR

Total RNA was extracted from primary cultured cardiomyocytes after reperfusion for 2 hours using TRIzol reagent (T9424, Sigma-Aldrich, USA). The absorbance at 260 nm and ratio at 260/280 nm were measured by a microplate spectrophotometer (Eon, BioTek, USA) to determine the concentration and purity of the extracted RNA. First-strand cDNA was generated by reverse transcription of 2 *μ*g total RNA using the PrimeScript™ RT kit (RR047A, TaKaRa Bio, Japan). The primers used to amplify the target gene are as follows: interleukin-18 (IL-18) forward: 5′-CAACCGCAGTAATACGGAGC-3′; IL-18 reverse: 5′-GATTCGTTGGCTGTTCGGTC-3′; interleukin-1*β* (IL-1*β*) forward: 5′-ACAGCAGCATCTCGACAAGAGC-3′; IL-1*β* reverse: 5′-CCACGGGCAAGACATAGGTAGC-3′; *β*-actin forward: 5′-ACAACCTTCTTGCAGCTCCTC-3′; *β*-actin reverse: 5′-CTGACCCATACCCACCATCAC-3′. After the cDNA was diluted 3 times, a mixed system of 20 *μ*l was prepared to complete the PCR reaction using the 7500 Real-Time PCR System (Applied Biosystems, USA). The results were analyzed according to the melt curve and the *Cт* value.

### 2.9. Western Blot

Briefly, each sample was equally quantified at 40/50 *μ*g of total protein and isolated with sodium dodecyl sulfate polyacrylamide gel electrophoresis (SDS-PAGE) (P1200, Solarbio, China) followed by transferring to the polyvinylidene fluoride (PVDF) (IPVH00010, Millipore, USA) membrane. The membranes were blocked with 3% bovine serum albumin (BSA) for 1 hour and then incubated with the primary antibody: gasdermin D (1 : 1000, 39754, Cell Signaling Technology, USA), IL-1*β* (1 : 1000, ab9722, Abcam, UK), phospho-NF-*κ*B p65 (1 : 1000, 3033, Cell Signaling Technology, USA), NF-*κ*B p65 (1 : 1000, 8242, Cell Signaling Technology, USA), phospho-I*κ*B*α* (1 : 1000, 2859, Cell Signaling Technology, USA), I*κ*B*α* (1 : 1000, 4812, Cell Signaling Technology, USA), and GAPDH (1 : 2000, 5174, Cell Signaling Technology, USA) at 4°C overnight. Then, the membranes were incubated with the secondary HRP-linked antibody (1 : 2000, 7074, Cell Signaling Technology, USA) at room temperature for 1 hour. The protein bands were scanned using a chemiluminescence imaging system (FluorChem FC3, ProteinSimple, USA) through the ECL developer, and the results were quantified by ImageJ.

### 2.10. Enzyme-Linked Immunosorbent Assay

The concentration of cTnI in serum of mice was measured according to the procedure of the Mouse Cardiac Troponin I Elisa Kit (SEKM-0153, Solarbio, China). The concentration of IL-18 in the cellular supernatants of primary cultured cardiomyocytes was measured by the ELISA kit (EK1173, Signalway Antibody, USA). Primary cultured cardiomyocytes were seeded in 96-well plates at a density of 1 × 10^4^~105 cells/well. After ischemia for 2 hours and reperfusion for 24 hours, testing was performed in accordance with the protocols contained in the ELISA kit. The absorbance of each well was detected at 450 nm using a microplate spectrophotometer (Eon, BioTek, USA). Then, the absorbance is converted to a concentration of cTnI or IL-18 according to the standard curve.

### 2.11. Statistical Analysis

All data were expressed as mean ± SEM and analyzed by SPSS v17.0 software (SPSS Inc., Chicago, IL, USA). Using one-way ANOVA analysis for comparison among more than two groups and using Levene's test for homogeneity of variance of each group. The least significant difference (LSD) test was employed to analyze the differences among multiple groups if equal variance is met; the Games-Howell test was performed if unequal variances are met. *p* < 0.05 was considered statistically significant.

## 3. Results

### 3.1. sRAGE Improved Cardiac Function following Myocardial Ischemia-Reperfusion Injury

**Figure 1:**
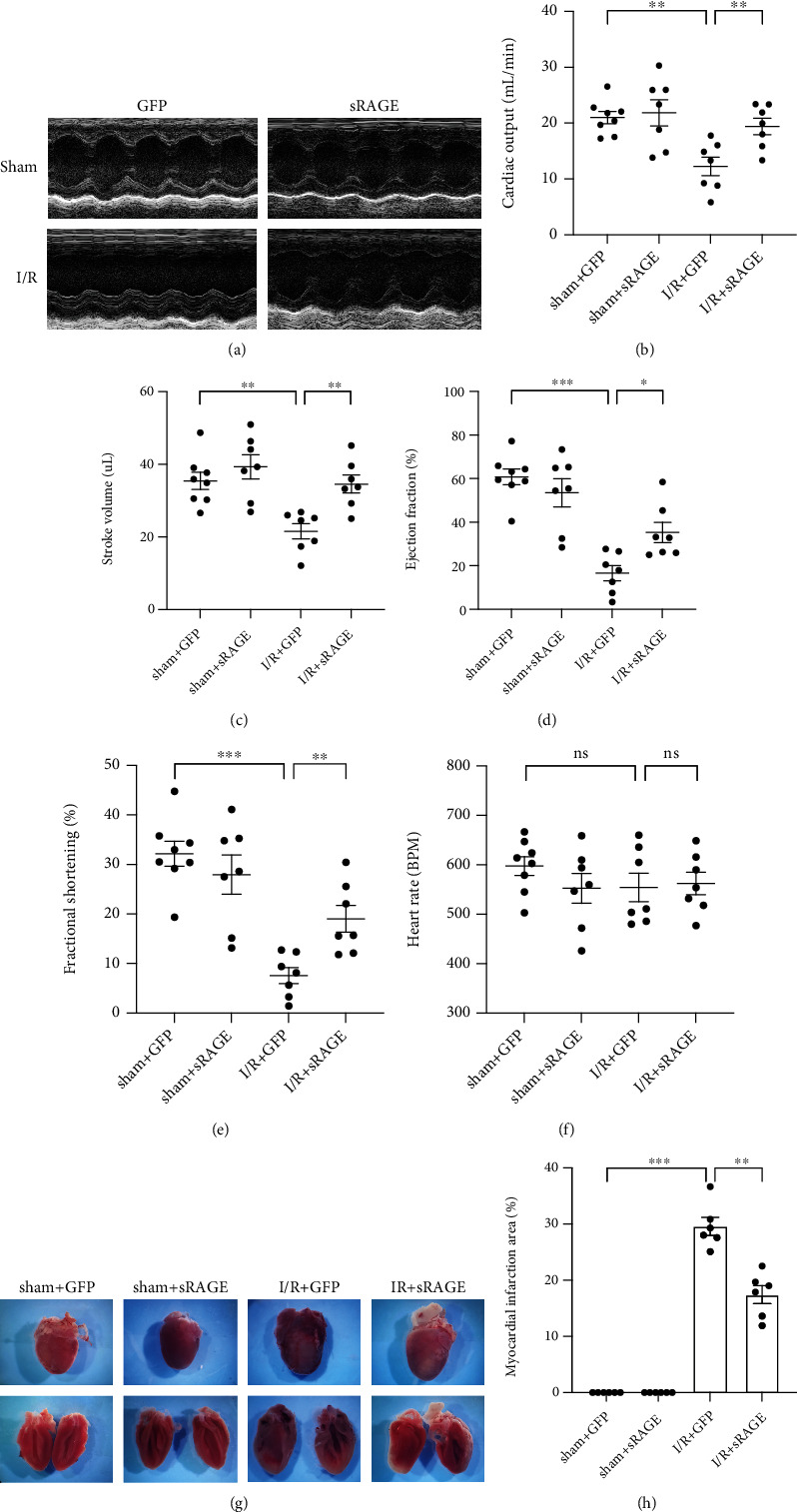
Effects of sRAGE on cardiac function and infarction size following myocardial ischemia-reperfusion in mice. (a) Representative M-mode echocardiography images of the long axis after ischemia for 30 minutes and reperfusion for 24 hours in mice. (b) Quantification of the left ventricular cardiac output (ml/min) of each group. (c) Quantification of the left ventricular stroke volume (*μ*l) of each group. (d) Quantification of the left ventricular ejection fraction (%) of each group. (e) Quantification of the left ventricular fractional shortening (%) of each group. (f) Heart rate (BPM) value of each group. (g) TTC staining pictures of the heart after I/R or sham operation. (h) Infarction area/total area of the sagittal plane of the left ventricle (%) of each group. Data are expressed as the mean ± SEM (*n* ≥ 6 mice per group). ^∗^*p* < 0.05; ^∗∗^*p* < 0.01; ^∗∗∗^*p* < 0.001; ns: no significance.

To evaluate the effect of sRAGE on myocardial ischemia-reperfusion, the I/R model in C57/BL6 mice was adopted. After reperfusion for 24 hours, the movement of the left ventricle anterior wall in the I/R group was significantly weakened than that in the sham group. However, the amplitude of anterior wall motion had a remarkable improvement with sRAGE treatment after I/R injury (Figure 1(a)). The CO was significantly decreased after I/R injury compared to that of the sham group (from 21.01 ± 3.04 ml/min to 12.26 ± 4.38 ml/min, *p* < 0.01; Figure 1(b)), which was reversed by sRAGE (reversed to 19.42 ± 3.85 ml/min, *p* < 0.01; Figure 1(b)). The same results were observed in SV (from 35.48 ± 6.81 *μ*l to 21.59 ± 5.52 *μ*l, *p* < 0.01; reverse to 34.59 ± 6.58 *μ*l, *p* < 0.01; Figure 1(c)), EF (from 60.82 ± 10.34% to 16.62 ± 9.27%, *p* < 0.001; reverse to 35.30 ± 12.40%, *p* < 0.05; Figure 1(d)), and FS (from 32.18 ± 7.14% to 7.57 ± 4.34%, *p* < 0.001; reverse to 19.03 ± 7.14%, *p* < 0.01; Figure 1(e)). At the same time, there was no statistical difference in HR among the groups (*p* > 0.05, Figure 1(f)), which supported the role of sRAGE in protecting cardiac function during myocardial ischemia-reperfusion. The results from TTC staining (Figure 1(g)) showed that the infarct size was significantly increased after I/R injury (up to 29.58 ± 3.96%, *p* < 0.001, Figure 1(h)) compared to that of the sham group, which was reversed by sRAGE (reversed to 17.44 ± 3.96%, *p* < 0.01, Figure 1(h)). These findings suggested that sRAGE had a cardioprotective effect on myocardial ischemia-reperfusion injury in vivo.

### 3.2. sRAGE Attenuated Apoptosis and Necrosis in the Myocardial Ischemia-Reperfusion Model

**Figure 2:**
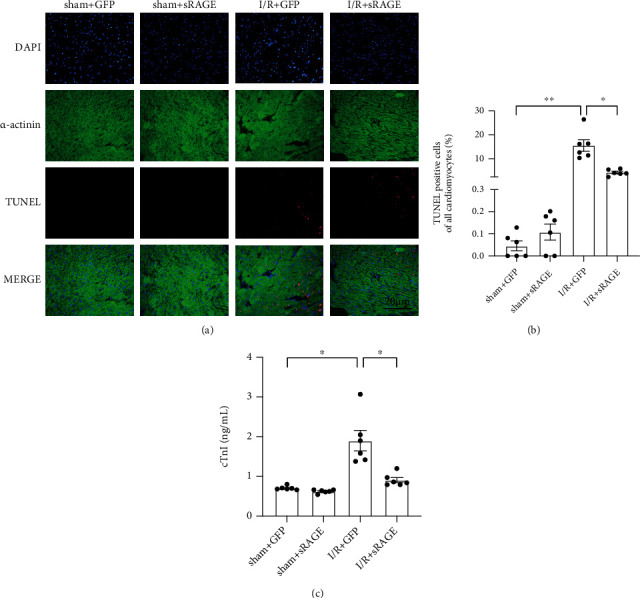
Effects of sRAGE on apoptosis and necrosis following myocardial ischemia-reperfusion in mice. (a) Representative TUNEL staining image in the myocardium. The target protein was shown in red, indicating apoptosis-positive cardiomyocyte. The scale bar is 20 *μ*m. (b) Quantitative statistics of TUNEL staining is shown above. (c) The detection of the serum cTnI level (ng/ml). Data are expressed as the mean ± SEM (*n* = 6 mice per group). ^∗^*p* < 0.05; ^∗∗^*p* < 0.01; ^∗∗∗^*p* < 0.001; ns: no significance.

When ischemia-reperfusion injury occurs, a variety of cell death processes are activated, of which apoptosis is an important part in myocardial injury. To further explore the function of sRAGE in I/R-induced myocardial injury, apoptosis was evaluated by TUNEL assay. The results (Figure 2(a)) showed that the apoptosis was significantly increased after I/R injury compared to that of the sham group (from 0.05 ± 0.06% to 15.51 ± 5.86%, *p* < 0.01, Figure 2(b)), which was reversed by sRAGE (reversed to 4.50 ± 2.00%, *p* < 0.05, Figure 2(b)). In addition, the effect of sRAGE on necrosis was also measured by cTnI (cardiac troponin I) in serum, which was elevated in the I/R + GFP group (from 0.71 ± 0.05 ng/ml to 1.90 ± 0.63 ng/ml, *p* < 0.05; Figure 2(c)) and then reversed by sRAGE (reversed to 0.91 ± 0.16 ng/ml, *p* < 0.05; Figure 2(c)). These data indicated that sRAGE had an inhibiting effect on apoptosis and necrosis after myocardial ischemia-reperfusion injury in vivo.

### 3.3. sRAGE Inhibited Pyroptosis during Myocardial Ischemia-Reperfusion

**Figure 3:**
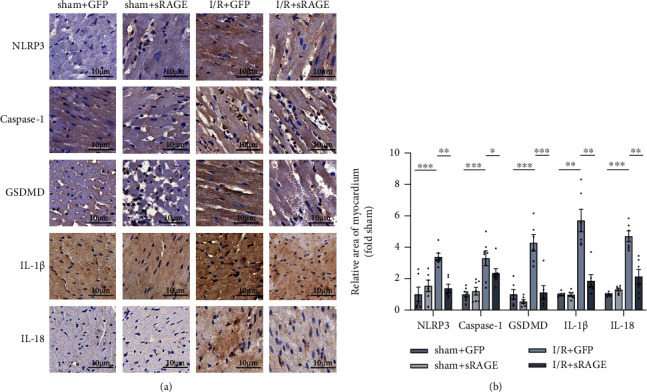
Effects of sRAGE on pyroptosis in the myocardium following myocardial ischemia-reperfusion injury. (a) Representative immunohistochemical image of pyroptosis-associated proteins in myocardium, including NLRP3, caspase-1, GSDMD, IL-1*β*, and IL-18. The target protein is shown in brown, indicating pyroptosis-positive cardiomyocyte. The scale bar is 10 *μ*m. (b) Quantitative statistics of immunohistochemical images is shown above. Data are expressed as the mean ± SEM (*n* ≥ 6 mice per group). ^∗^*p* < 0.05; ^∗∗^*p* < 0.01; ^∗∗∗^*p* < 0.001; ns: no significance.

To verify the role of sRAGE on NLRP3 inflammasome-mediated pyroptosis in myocardial ischemia-reperfusion, immunohistochemical staining was performed. Compared with the sham group, the protein expression levels of NLRP3, casepase-1, GSDMD, IL-1*β*, and IL-18 were significantly increased in the I/R group, which were reversed by sRAGE (Figure 3). These findings confirmed that sRAGE inhibited NLRP3 inflammasome-mediated pyroptosis during myocardial ischemia-reperfusion.

### 3.4. sRAGE Suppressed the Transcription of IL-1*β* and IL-18 in Primary Cultured Cardiomyocytes following Ischemia-Reperfusion Injury

**Figure 4:**
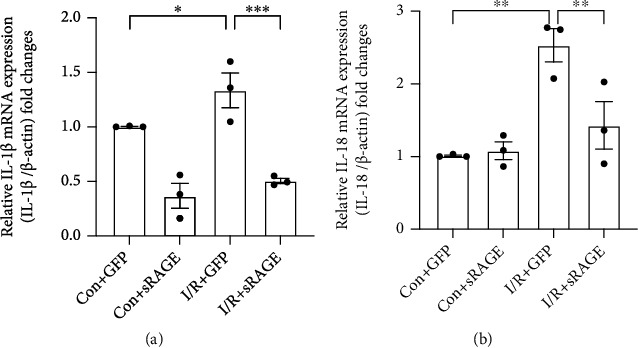
Effects of sRAGE on the transcription of IL-1*β* mRNA and IL-18 mRNA in primary cultured cardiomyocytes following ischemia-reperfusion injury. I/R groups were subjected to ischemia for 2 hours and reperfusion for 2 hours. (a) Quantitative RT-PCR of interleukin-1*β* mRNA expression in cardiomyocytes. (b) Quantitative RT-PCR of interleukin-18 mRNA expression in cardiomyocytes. *β*-Actin was used as the reference gene. Data are expressed as mean ± SEM (*n* = 3 replicates). ^∗^*p* < 0.05; ^∗∗^*p* < 0.01; ^∗∗∗^*p* < 0.001; ns: no significance.

To investigate the transcription of inflammatory factors in primary cultured cardiomyocytes after ischemia-reperfusion, IL-1*β* and IL-18 mRNA levels were measured by RT-PCR assay. The results showed that the I/R group exhibited higher expression levels of IL-1*β* compared with the control group (*p* < 0.05, Figure 4(a)), which was reversed by sRAGE (*p* < 0.001, Figure 4(a)). The same results were observed in IL-18 (*p* < 0.01, Figure 4(b)). The data suggested that sRAGE suppressed the transcription of IL-1*β* and IL-18 in primary cultured cardiomyocytes following ischemia-reperfusion injury.

### 3.5. sRAGE Decreased I/R-Induced Pyroptosis in Primary Cultured Cardiomyocytes

**Figure 5:**
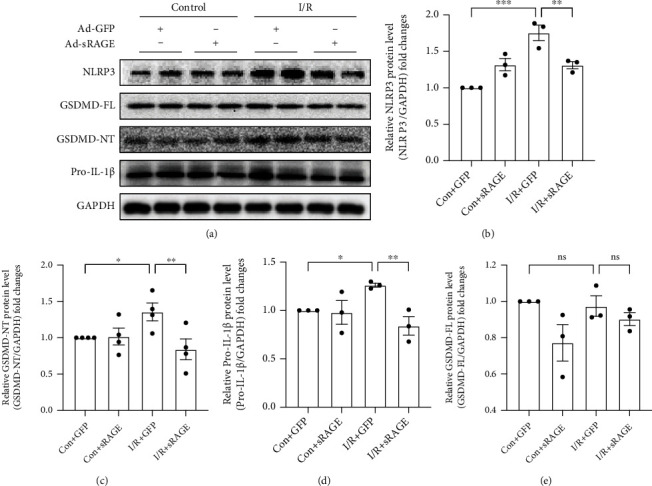
Effects of sRAGE on the expression of pyroptosis-associated proteins in primary cultured cardiomyocytes following ischemia-reperfusion injury. I/R groups were subjected to ischemia for 2 hours and reperfusion for 24 hours. (a) Representative images of Western blot for pyroptosis-associated proteins. The histogram depicts the quantitative densitometry analysis of Western blot data. (b) Western blot analysis of NLRP3 protein expression. (c) Western blot analysis of the NH2-terminal cleaved GSDMD protein level. (d) Western blot analysis of the pro-IL-1*β* protein level. (e) Western blot analysis of the full-length GSDMD protein level. GAPDH was used as the internal reference control. Data are expressed as mean ± SEM (*n* ≥ 3 replicates). ^∗^*p* < 0.05; ^∗∗^*p* < 0.01; ^∗∗∗^*p* < 0.001; ns: no significance.

**Figure 6:**
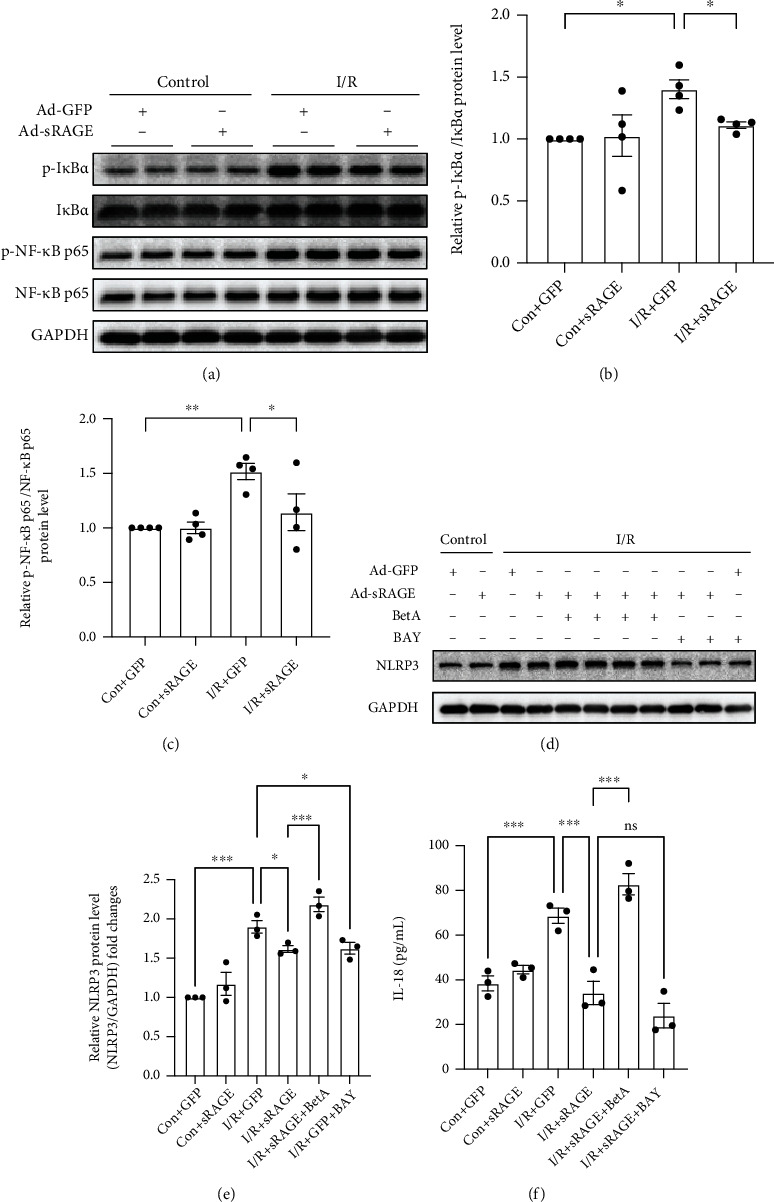
Effects of sRAGE on the NF-*κ*B pathway in primary cardiomyocytes against I/R-induced pyroptosis. (a) Representative images of Western blot for the phosphorylation level of I*κ*B*α* and NF-*κ*B p65 proteins. The histogram depicts the quantitative densitometry analysis of Western blot data. (b) Western blot analysis of the phosphorylation level of I*κ*B*α*. (c) Western blot analysis of the phosphorylation level of NF-*κ*B p65. GAPDH was used as the loading control. (d) Representative images of Western blot for the NLRP3 level. I/R groups were subjected to ischemia for 2 hours and reperfusion for 24 hours. (e) Western blot analysis of the NLRP3 level. (f) The level of IL-18 cytokines excreted by primary cultured cardiomyocytes within 24 hours of reperfusion. Data are expressed as mean ± SEM (*n* ≥ 3 replicates). BetA: betulinic acid; BAY: BAY117082; ^∗^*p* < 0.05; ^∗∗^*p* < 0.01; ^∗∗∗^*p* < 0.001; ns: no significance.

To figure out whether the effect of sRAGE is direct on cardiomyocytes or not, primary cultured cardiomyocytes were adopted. Compared with the control group, NLRP3, GSDMD-NT, and pro-IL-1*β* increased after reperfusion for 24 hours (*p* < 0.05, Figures 5(a)–5(d)), while sRAGE reduced the I/R-induced protein enhancement (*p* < 0.01, Figures 5(a)–5(d)). Western blot analysis confirmed the NH2-terminal cleavage of GSDMD-FL, which is the marker of pyroptosis [13]. However, there was no significant difference in GSDMD-FL (*p* > 0.05, Figure 5(e)) between the control group and I/R group. As pyroptosis is bound to cause IL-18 leakage, the concentration of IL-18 in the cell supernatant was tested after reperfusion for 24 hours. In contrast to the control group, the concentration of IL-18 in the I/R group was obviously increased, which was reversed by sRAGE (*p* < 0.001, Figure 6(f)). These results suggested that sRAGE directly acted on cardiomyocytes to suppress I/R-induced pyroptosis.

### 3.6. NF-*κ*B Pathway Mediated the Protective Effects of sRAGE on I/R-Induced Pyroptosis in Primary Cultured Cardiomyocytes

To evaluate the activation of the NF-*κ*B pathway in the protective effects of sRAGE against I/R-induced pyroptosis in primary cultured cardiomyocytes, Western blot assay was performed. The results showed that the phosphorylation levels of I*κ*B and NF-*κ*B were increased in the I/R group compared with the control group, which was reversed by sRAGE (Figures 6(a)–6(c)). To further verify the role of the NF-*κ*B pathway on pyroptosis, an NF-*κ*B activator, betulinic acid (20 *μ*M), and an NF-*κ*B inhibitor, BAY117082 (10 *μ*M) were used [31, 32].

As the activation of pyroptosis is mediated by NLRP3 inflammasome, the expression of NLRP3 was assessed by Western blot. NLRP3 levels of the I/R group increased significantly compared to these of the control group, which were reversed by sRAGE (Figures 6(d) and 6(e)). Betulinic acid reversed the inhibitory effect of sRAGE on the expression of NLRP3 (*p* < 0.001, Figures 6(d) and 6(e)), meanwhile the inhibitor group had the same effect as the sRAGE group (*p* > 0.05, Figures 6(d) and 6(e)).

Likewise, the supernatant of cardiomyocytes was collected and then measured by the ELISA kit. IL-18 in the supernatant of the cardiomyocytes in the I/R group increased significantly compared to that in the control group (from 38.46 ± 5.72 pg/ml to 68.68 ± 5.92 pg/ml, *p* < 0.001, Figure 6(f)), which was reversed by sRAGE (reversed to 34.20 ± 9.01 pg/ml, *p* < 0.001, Figure 6(f)). Betulinic acid reversed the inhibitory effect of sRAGE on the secretion of IL-18 (from 34.20 ± 9.01 pg/ml to 82.79 ± 8.26 pg/ml, *p* < 0.001, Figure 6(f)), while there was no significant statistical difference between the inhibitor group and the I/R + sRAGE group (from 34.20 ± 9.01 pg/ml to 24.04 ± 9.46 pg/ml, *p* > 0.05, Figure 6(f)).

The results showed that the activation of NF-*κ*B could eliminate the inhibitory effect of sRAGE on pyroptosis, which leads to the inhibitory effect of sRAGE on I/R-induced pyroptosis in primary cardiomyocytes through the NF-*κ*B pathway.

## 4. Discussion

Acute myocardial infarction (AMI) remains a common cause of hospitalization and death worldwide, whose treatment in clinical would inevitably cause reperfusion injury that exacerbates the local aseptic inflammatory response [1, 2]. Some researchers have proposed that reperfusion injury accounts for 50% of the total myocardial injury [2, 8]. Therefore, if measures are taken to alleviate I/R injury, the prognosis of patients could be further improved [2]. Pyroptosis is a recently proposed programmed cell death pattern associated with inflammatory response, which plays an important role in the process of ischemia-reperfusion injury [11, 14].

The present study demonstrated that sRAGE protected the heart from ischemia-reperfusion injury via decreasing pyroptosis in the cardiomyocytes in addition to apoptosis and necrosis, which were related to the NF-*κ*B pathway-activated molecules. The results showed that sRAGE would not only improve cardiac function and diminish the infarction size but also reduce the occurrence of apoptosis, necrosis, and pyroptosis in I/R-treated myocardium. Meanwhile, sRAGE also reduced the levels of pyroptosis-related proteins in cardiomyocytes, such as NLRP3, GSDMD-NT, IL-1*β*, and IL-18, which were related to the NF-*κ*B pathway.

First of all, the results of echocardiography showed that sRAGE did improve the cardiac function after I/R injury, including CO, SV, EF, and FS (Figures 1(a)–1(e)). In addition, it was observed that sRAGE did decrease the infarction size after I/R injury as shown in TTC staining (Figure 1(g)), which were related to apoptosis and necrosis in myocardium as observed in TUNEL and serum cTnI (Figure 2). These results are consistent with previous studies which showed that sRAGE protected the heart from I/R injury [23–26, 33]. Meanwhile, pyroptosis has been reported to be one of the important cell deaths in myocardial ischemia-reperfusion [11]; hence, it is supposed that decreasing pyroptosis may be one of the ways to attenuate I/R injury by sRAGE.

Therefore, the role of sRAGE on pyroptosis was explored in cardiac tissue after suffering from I/R injury. As shown in immunohistochemical staining (Figure 3(a)), sRAGE decreased the expression of pyroptosis-associated proteins, including NLRP3, GSDMD, caspase-1, IL-1*β*, and IL-18 (Figure 3(b)), which leads to the conclusion that pyroptosis was decreased by sRAGE in I/R-treated myocardium. These findings suggested that pyroptosis might be one of the mechanisms of cell deaths protected by sRAGE in myocardial ischemia-reperfusion injury.

To verify whether sRAGE decreases the pyroptosis by directly acting on cardiomyocytes or not, primary cultured cardiomyocytes were adopted. As it is expected, the results from Western blot and RT-PCR assay showed that the expression of pyroptosis-associated proteins including NLRP3, IL-18, IL-1*β*, and GSDMD-NT which stands for the end-stage executor of pyroptosis [13, 34] was decreased by sRAGE after I/R injury in primary cultured cardiomyocytes (Figures 4 and 5). Therefore, it is considered that sRAGE acts directly on cardiomyocytes in protecting the heart from I/R injury.

As is well known, the first step of pyroptosis is the activation of the inflammasome in which the “priming” step is necessary for cardiomyocytes [11, 12]. Due to the report that the NF-*κ*B pathway mediated the transcription of NLRP3, which plays an important role in the “priming” step of NLRP3 inflammasome activation [16], the effect of sRAGE on the NF-*κ*B pathway was observed. The results from Western blot showed that the phosphorylation of I*κ*B and NF-*κ*B was suppressed by sRAGE during I/R injury in cardiomyocytes (Figures 6(a)–6(c)), which mediated the expression of NLPR3, because activation of NF-*κ*B increased the expression of NLRP3 and vice versa (Figures 6(d) and 6(e)). As the expression of IL-18, which will be released based on the occurrence of pyroptosis, is also activated through the NF-*κ*B pathway [11, 35, 36], IL-18 in the cell supernatant was tested by ELISA assay. The results showed that the expression of IL-18 was decreased by sRAGE in the primary cultured cardiomyocytes after I/R injury, which was reversed by betulinic acid, the activator of the NF-*κ*B pathway, but not affected by the inhibitor of the NF-*κ*B pathway (Figure 6(f)). Thus, the NF-*κ*B pathway was supposed to be the upstream of pyroptosis. Therefore, it is reasonable to assume that sRAGE might protect the cardiomyocytes from pyroptosis besides apoptosis and necrosis in ischemia-reperfusion injury via inhibiting the NF-*κ*B pathway.

However, results from Western blot assay showed that GSDMD was not elevated in cardiomyocytes after I/R treatment (Figure 5(e)), which was inconsistent with in vivo results in hearts (Figure 3). As GSDMD expression was reported to be activated in noncardiomyocytes during I/R injury [37], it is supposed that the expression of GSDMD in the heart may either be in noncardiomyocytes or stimulated by noncardiomyocytes during I/R.

## 5. Conclusions

In summary, this study revealed for the first time that sRAGE protected the heart from I/R injury via decreasing pyroptosis which was mediated by inhibiting the activation of NLRP3 inflammasome through the NF-*κ*B pathway. As a promising therapeutic target for I/R injury, sRAGE is worthy of more detailed and rigorous investigation.

## Data Availability

The experimental data used to support the findings of this study are available from the corresponding author upon request.

## Abbreviations

IRI: Ischemia-reperfusion injury
I/R: Ischemia-reperfusion
sRAGE: Soluble receptor for advanced glycation end-products
RAGE: Receptor for advanced glycation end-products
NLRP3: NLR family pyrin domain containing 3
ASC: Apoptosis-associated speck-like protein containing a caspase recruitment domain
GSDMD: Gasdermin D
GSDMD-FL: Full-length gasdermin D
GSDMD-NT: NH2-terminal cleaved-gasdermin D
IL-1*β*: Interleukin-1*β*
IL-18: Interleukin-18
AMI: Acute myocardial infarction
AAV: Adeno-associated virus
LAD: Left anterior descending
HR: Heart rate
EF: Ejection fraction
FS: Fractional shortening
CO: Cardiac output
SV: Stroke volume
TTC: 2,3,5-Triphenyltetrazolium chloride
SD: Sprague-Dawley
PBS: Phosphate buffer saline
DMEM-F12: Dulbecco's modified Eagle's medium/nutrient mixture F-12 ham
RT-PCR: Reverse transcription-polymerase chain reaction
SDS-PAGE: Sodium dodecyl sulfate polyacrylamide gel electrophoresis
PVDF: Polyvinylidene fluoride
BSA: Bovine serum albumin
ELISA: Enzyme-linked immunosorbent assay
LSD: Least significant difference
BetA: Betulinic acid
BAY: BAY117082

## Supplementary Materials

Supplementary Materials Supplemental materials include the extended schematic diagram of the signal transduction pathways of pyroptosis in myocardial I/R injury.
